# Transactions of the Twenty-ninth Annual Meeting of the Medical Society of Virginia, Together with the President’s Annual Address and the Constitution of the Society

**Published:** 1852-11

**Authors:** 


*Transactions of the Twenty-Ninth Annual Meeting of the Medical Society of Virginia, together with the President's Annual Address and the Constitution of the Society.* Richmond, 1852.

The Transactions of the Medical Society of Virginia, which met at Richmond, in April last, appear to have been of considerable interest. The subject of medical education, and, in particular, the establishment by law of a State Board of Medical Examiners, occupied much of the attention of the Society, and elicited animated discussion. It would seem that the Society leaned so far to the interest of the Schools, as to refuse to recommend to the

Legislature the *unqualified* establishment of a State Board of Examiners, but suggested "the propriety of requiring as a condition precedent to every examination, that the applicant shall have been graduated in medicine, or shall have attended two full courses of lectures in some respectable medical College." Drs. Tucker, Atkinson, Haskins, Worsham and others took part in the debate on this question.

The Address of the distinguished President of the Society, Dr. Beverley R. Wellford, of Fredericksburg, is a very forcible argument in favor of reform in our system of medical education. Dr. Wellford has been one of the pioneers in this cause, and we are gratified to find him still lending his high name and earnest eloquence to its advancement.

